# *Alx1* Deficient Mice Recapitulate Craniofacial Phenotype and Reveal Developmental Basis of *ALX1*-Related Frontonasal Dysplasia

**DOI:** 10.3389/fcell.2022.777887

**Published:** 2022-01-21

**Authors:** Paul P. R. Iyyanar, Zhaoming Wu, Yu Lan, Yueh-Chiang Hu, Rulang Jiang

**Affiliations:** ^1^ Division of Developmental Biology, Cincinnati Children’s Hospital Medical Center, Cincinnati, OH, United States; ^2^ Division of Plastic Surgery, Cincinnati Children’s Hospital Medical Center, Cincinnati, OH, United States; ^3^ Departments of Pediatrics and Surgery, University of Cincinnati College of Medicine, Cincinnati, OH, United States

**Keywords:** cleft palate, craniofacial development, frontonasal dysplasia, microphthalmia, neural crest, orofacial cleft, periocular mesenchyme, alx1

## Abstract

Loss of ALX1 function causes the frontonasal dysplasia syndrome FND3, characterized by severe facial clefting and microphthalmia. Whereas the laboratory mouse has been the preeminent animal model for studying developmental mechanisms of human craniofacial birth defects, the roles of ALX1 in mouse frontonasal development have not been well characterized because the only previously reported *Alx1* mutant mouse line exhibited acrania due to a genetic background-dependent failure of cranial neural tube closure. Using CRISPR/Cas9-mediated genome editing, we have generated an *Alx1-deletion* mouse model that recapitulates the FND craniofacial malformations, including median orofacial clefting and disruption of development of the eyes and alae nasi*. In situ* hybridization analysis showed that *Alx1* is strongly expressed in frontonasal neural crest cells that give rise to periocular and frontonasal mesenchyme. *Alx1* ^*del/del*^ embryos exhibited increased apoptosis of periocular mesenchyme and decreased expression of ocular developmental regulators *Pitx2* and *Lmxb1* in the periocular mesenchyme, followed by defective optic stalk morphogenesis. Moreover, *Alx1* ^*del/del*^ embryos exhibited disruption of frontonasal mesenchyme identity, with loss of expression of *Pax7* and concomitant ectopic expression of the jaw mesenchyme regulators *Lhx6* and *Lhx8* in the developing lateral nasal processes. The function of ALX1 in patterning the frontonasal mesenchyme is partly complemented by ALX4, a paralogous ALX family transcription factor whose loss-of-function causes a milder and distinctive FND. Together, these data uncover previously unknown roles of ALX1 in periocular mesenchyme development and frontonasal mesenchyme patterning, providing novel insights into the pathogenic mechanisms of *ALX1*-related FND.

## Introduction

Frontonasal dysplasia (FND), also known as median cleft face syndrome, is a group of congenital craniofacial disorders characterized by ocular hypertelorism, midline facial cleft affecting the nose and/or upper lip and palate, broad and flattened nasal bridge, notching or clefting of the nasal alae, and is sometimes associated with anterior cranium bifidum and other malformations (Wu et al., 2007; Kayserili et al., 2009; Twigg et al., 2009; Farlie et al., 2016). Most of the bones, cartilages, and other connective tissues in the face are derived from a transient embryonic cell population called the cranial neural crest cells (CNCCs), which arise at the anterior neural plate border in human embryos in the third week of gestation, corresponding to about embryonic day (E) 8.0 in mice (Jiang et al., 2000; O’Rahilly and Müller, 2007; Yoshida et al., 2008; Zalc et al., 2021). The first group of CNCCs delaminate from the region lateral to the prospective forebrain and anterior midbrain, migrate ventrally to surround the ventral forebrain and interact with both neural and surface ectoderm to form the embryonic frontonasal prominence (FNP) by mid-fourth week of human gestation, corresponding to about E9.0 in mice (Jiang et al., 2006). The second group of CNCCs delaminate at the posterior midbrain and anterior hindbrain level and migrate ventrally to interact with the surface ectoderm to form the maxillary and mandibular processes. Thus, the initial facial primordia, observed in four-week-old human embryos and E9.5 mouse embryos, consist of the single FNP located rostrally to the primitive mouth, a pair of maxillary processes (MxP) flanking and a pair of mandibular processes at the caudal boundary of the primitive mouth (Jiang et al., 2006). As facial development continues, the FNP gives rise to paired medial nasal processes (MNP) and lateral nasal processes (LNP) flanking each of the nasal pits. Formation of the intact upper lip involves extensive directional growth and subsequent fusion of the MNP, LNP, and MxP as well as merging of the nasal processes to fill the facial midline (Jiang et al., 2006). Thus, the causes of FND are complex and could result from genetic and/or environmental perturbations of CNCC migration, proliferation, survival, differentiation, or the lip fusion processes.

Whilst most FND cases occur sporadically with unknown etiology, homozygous loss-of-function mutations in *ALX1*, *ALX3*, or *ALX4*, have been associated with distinct recessive FNDs. Disruption of *ALX1* causes severe facial clefting and microphthalmia in FND3 patients, whereas loss of function mutations in *ALX3* and *ALX4* underlie the milder FND1 and FND2 syndromes, respectively (Kayserili et al., 2009; Twigg et al., 2009; Uz et al., 2010; Pini et al., 2020). A mutation altering the splice acceptor of the fourth exon of *ALX1* has been associated with a milder form of FND3, with patients displaying ptosis (droopy upper eyelid), broad nasal root, short and wide nasal bridge, bifid or depressed nasal tip and anteverted nares (Ullah et al., 2017). The *Alx* genes encode homeodomain-containing transcription factors that are expressed in partly overlapping patterns during craniofacial development (Beverdam and Meijlink, 2001; McGonnell et al., 2011; Dee et al., 2013). Gene knockout studies of each of the *Alx* genes in mice had been reported prior to the discovery of *ALX* gene mutations in human FND patients. An *Alx1* gene-knockout mouse line, in which the first three exons of the *Alx1* gene were replaced with a *neomycin* expression cassette (*Alx1* ^*tm1Crm*^ is the official name of that *Alx1* gene-knockout allele), exhibited aberrant apoptosis of the embryonic forebrain mesenchyme and failure of cranial neural tube closure in homozygous mutant mice (Zhao et al., 1996). Mice lacking *Alx3* function did not have an obvious defect in craniofacial development, whereas mice lacking *Alx4* function exhibited multiple developmental defects, including limb malformations and ventral body wall defects but only mild frontonasal defect with variable open eyelid at birth (Qu et al., 1997; Beverdam et al., 2001). In addition, a severe midline nasal clefting phenotype has been reported in mice carrying three to four disrupted alleles of *Alx3* and *Alx4* together or the *Alx1* ^*tm1Crm*/*+*^ *Alx4* ^*−/−*^ genotype (Qu et al., 1999; Beverdam et al., 2001). Although Zhao et al. (1996) indicated that the penetrance of neural tube defect in *Alx1* ^*tm1Crm*/*tm1Crm*^ mouse embryos was genetic background dependent, with about 65% of the *Alx1* ^*tm1Crm*/*tm1Crm*^ embryos in the B6/129 hybrid background displayed neural tube defect compared to 100% penetrance in the 129/SvEv inbred background (Zhao et al., 1996), no specific analysis of frontonasal development in the *Alx1* ^*tm1Crm*/*tm1Crm*^ mice, with or without neural tube defect, has been reported. Several laboratories have studied ALX1 function in craniofacial development using zebrafish models. Dee et al. (2013) reported that morpholino knockdown of *alx1* function in zebrafish embryos resulted in defective frontonasal neural crest cell migration and catastrophic failure of facial cartilage formation (Dee et al., 2013). However, Pini et al. (2020) showed that genetic loss of *alx1* function did not affect the development and viability of the majority of homozygous zebrafish mutants, with only a subtle deformity in facial cartilages detected in about 5% of the homozygous *alx1* mutant fish (Pini et al., 2020). Mitchell et al. (2021) independently generated and analyzed *alx1* loss-of-function mutant zebrafish lines and confirmed that ALX1 function is not essential for craniofacial development in zebrafish (Mitchell et al., 2021). Altogether, although there have been several genetic studies of ALX1 function in mice and zebrafish, the cellular and molecular mechanisms mediating ALX1 function in frontonasal development remain unresolved.

In this study, we generated *Alx1*-deficient mice using CRISPR/Cas9-mediated genome editing in the C57BL/6N inbred strain and found that *Alx1* mutant mice recapitulated craniofacial defects found in *ALX*-related FND patients, including frontonasal malformations, notching of the upper lip, cleft palate, and eye morphogenesis defects (Farlie et al., 2016). In contrast to the defective CNCC migration reported in zebrafish *alx1* morphants (Dee et al., 2013), CNCC migration to the FNP and pharyngeal arches occurred normally in the *Alx1* mutant mouse embryos. Further analyses identified novel roles of ALX1 in patterning the frontonasal and periocular mesenchyme, revealing a crucial role for ALX1 in determining the identity of the frontonasal neural crest-derived LNP mesenchyme and in patterning the midface.

## Results

### Mice Lacking *Alx1* Function Recapitulate Craniofacial and Ocular Defects in ALX-Related FND Patients

**FIGURE 1:**
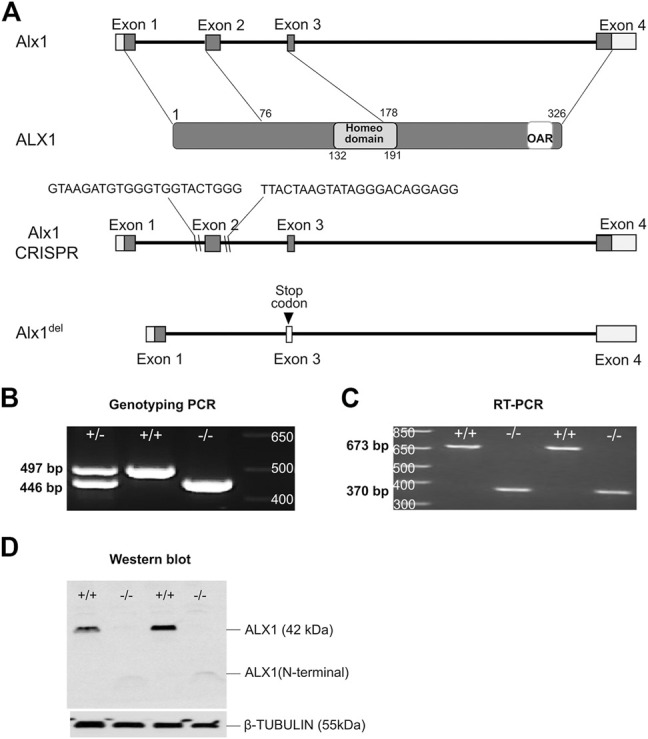
Generation of *Alx1*-deletion mice using CRISPR/Cas9-mediated genome editing. **(A)** Schematics of the strategy for generating *Alx1* ^*del/+*^ mice using the CRISPR/Cas9 technology. The top row shows the genomic organization of the mouse *Alx1* locus. Exons 1–4 are boxed with coding regions filled in grey color and the domains of ALX1 protein represented in the second row. Lines point to the exons of the *Alx1* gene with the corresponding region in the ALX1 protein. The DNA-binding Homeodomain and the OAR domain, a conserved domain in the C-terminal region of several paired-like homedomain proteins including *Drosophila* Orthopedia and Aristaless and vertebrate Rax, are marked. The locations of sgRNA target sequences are indicated in intron-1 and intron-2 regions, respectively, in the schematic in the third row, whereas the fourth row depicts the *Alx1* ^*del*^ allele that lacks exon-2 and flanking sequences in between the two sgRNA target sites. The exon-2 deletion results in a frameshift and premature translational STOP codon in the exon-3 sequence. **(B)** PCR genotyping of the embryos from the intercross of heterozygous *Alx1* ^*del/+*^ mice. The amplicons from wildtype and *Alx1* ^*del*^ alleles are 497 bp and 446 bp, respectively. **(C)** RT-PCR analysis using primers from exon1 to exon4, respectively, reveals a wildtype amplicon of 673 bp and an *Alx1* ^*del*^ allele-specific product of 370 bp. **(D)** Western blot analysis using a polyclonal anti-ALX1 antibody raised against the full-length ALX1 protein reveals a lack of the full-length ALX1 protein in the *Alx1* ^*del/del*^ embryos. *Alx1* ^*del/del*^ embryos produced a truncated N-terminal product of around 10.5 kDa *+/-*, *Alx1* ^*del/+*^; *+/+*, wildtype; *−/−*, *Alx1* ^*del/del*^.

To investigate the mechanism involving ALX1 in frontonasal development, we generated a new mutant mouse line carrying a deletion of exon-2 of the *Alx1* gene (*Alx1* ^*del*^) using CRISPR/Cas9-mediated genome editing in the C57BL/6N inbred mice (Figures 1A–C). Sequencing of RT-PCR products from *Alx1* ^*del/+*^ and *Alx1* ^*del/del*^ embryos confirmed that the *Alx1* ^*del*^ allele produced mutant mRNAs from splicing exon-1 to exon-3, which led to a frame-shift and is predicted to produce a truncated protein product containing only the N-terminal region of the ALX1 protein lacking the homeodomain and the C-terminal Aristaless domain (Brouwer et al., 2003). Indeed, western blot analysis confirmed that the *Alx1* ^*del/del*^ embryos lacked full-length ALX1 protein and only produced a truncated product that was expressed at low levels but still detectable using the polyclonal anti-ALX1 antibody (Figure 1D).

**FIGURE 2:**
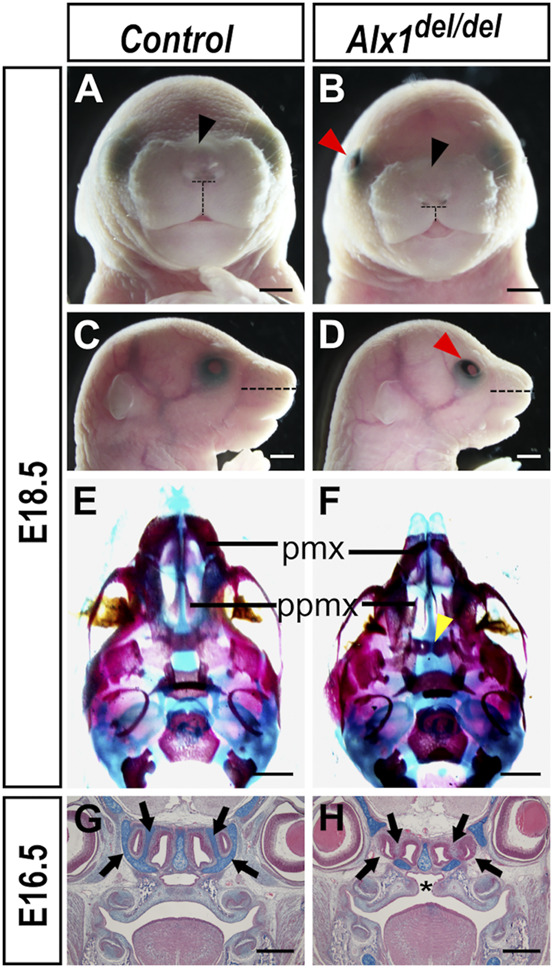
Craniofacial defects in the *Alx1* ^*del/del*^ embryos. **(A–D)** Whole mount frontal **(A, B)** and lateral **(C, D)** views of control **(A, C)** and *Alx1* ^*del/del*^ **(B, D)** embryos at E18.5. *Alx1* ^*del/del*^ embryos exhibit open eyelid [red arrowhead in **(B, D)**], shorter snout [compare horizontal dashed lines in **(C, D)**], wider nasal bridge [black arrowhead in **(B)**] and notching of the upper lip. Dashed horizontal lines in A and B mark the inter-nostril distance, whereas vertical dashed lines mark the length of the philtrum for measurements in Supplementary Figure S1. **(E, F)** Palatal view of the head skeleton preparations of E18.5 control **(E)** and *Alx1* ^*del/del*^ **(F)** embryos. *Alx1* ^*del/del*^ embryos exhibit hypoplastic premaxilla, malformed palatal processes, and hypoplastic presphenoid bone [yellow arrowhead in **(F)**]. **(G, H)** Frontal sections of control **(G)** and *Alx1* ^*del/del*^ **(H)** embryo heads at E16.5. Asterisk in H marks the cleft palate. Black arrows point to the lateral nasal cartilages in the wildtype control **(G)** and the defective nasal cartilages in the *Alx1* ^*del/del*^ embryos **(H)**. Scale bars in **(A–F)** and **(G, H)** are 500 µm and 1 mm, respectively. pmx, premaxilla; ppmx, palatal process of the maxillary bone.

*Alx1* ^*del/+*^ mice appeared indistinguishable from wildtype littermates. Examination of pups and fetuses from *Alx1* ^*del/+*^ mice intercrosses revealed that *Alx1* ^*del/del*^ pups exhibited shortened snout, flattened nasal bridge with increased distance between the nostrils, short philtrum, and midline notching of the upper lip (Figures 2A–D, *n* = 6 for each genotype; and Supplementary Figure S1). *Alx1* ^*del/del*^ pups were born alive but died soon after, most likely due to the cleft palate defect (*n* = 4 for each genotype examined by serial frontal sections at E16.5). In addition, 35 out of 50 E18.5 or newborn *Alx1* ^*del/del*^ pups examined showed open eyelid unilaterally (Figures 2B,D). Analysis of skeletal preparations revealed that *Alx1* ^*del/del*^ embryos had hypoplastic premaxilla and presphenoid bone, and malformed palatal processes (Figures 2E,F) (*n* = 5 for each genotype). Histological analyses of E16.5 *Alx1* ^*del/del*^ embryos and control littermates showed that, in addition to cleft palate defect, the *Alx1* ^*del/del*^ embryos had disruptions of nasal cartilages (Figures 2G,H) (*n* = 4 for each genotype). No neural tube defect has been detected in over 200 *Alx1* ^*del/del*^ embryos analyzed in the C57BL/6N background.

We outcrossed the *Alx1* ^*del/+*^ mice to 129/S6 inbred mice, a subline derived from the original 129/SvEv inbred strain (Simpson et al., 1997) that was used to analyze the *Alx1* ^*tm1Crm*^ allele (Zhao et al., 1996), for two generations and then intercrossed the N2 *Alx1* ^*del/+*^ male and female mice to analyze *Alx1* ^*del/del*^ pups in the 129 X C57BL/6 hybrid background. Only 5 of 48 (∼10%) *Alx1* ^*del/del*^ embryos harvested from E16.5 to E18.5 in this hybrid background displayed an exencephaly phenotype (Supplementary Figures S2A–C). While the frequency of *Alx1* ^*del/del*^ embryos exhibiting anterior neural tube defect in this study is low, these results confirm that loss of *Alx1* function causes a genetic background-dependent defect in anterior neural tube closure in mice. The *Alx1* ^*del/del*^ embryos with exencephaly showed more severe ocular defect but fused secondary palate (*n* = 5), whereas cleft palate was observed in all E16.5 or older *Alx1* ^*del/del*^ embryos that did not have exencephaly and subjected to examination of palate morphology (*n* = 16) (Supplementary Figures S2E,F). All *Alx1* ^*del/del*^ pups in the 129 X C57BL/6 hybrid background exhibited similar frontonasal defects as in the C57BL/6N inbred background, including flattened nasal bridge, midline notching of the upper lip, and disruption of nasal cartilages (Supplementary Figures S2A–F). These midfacial defects in *Alx1* ^*del/del*^ mice closely recapitulate the midfacial developmental defects in *ALX*-related patients.

### *Alx1* is Expressed in the CNCC-Derived Mesenchyme of the LNP and MNP as Well as in the Periocular Mesenchyme

**FIGURE 3:**
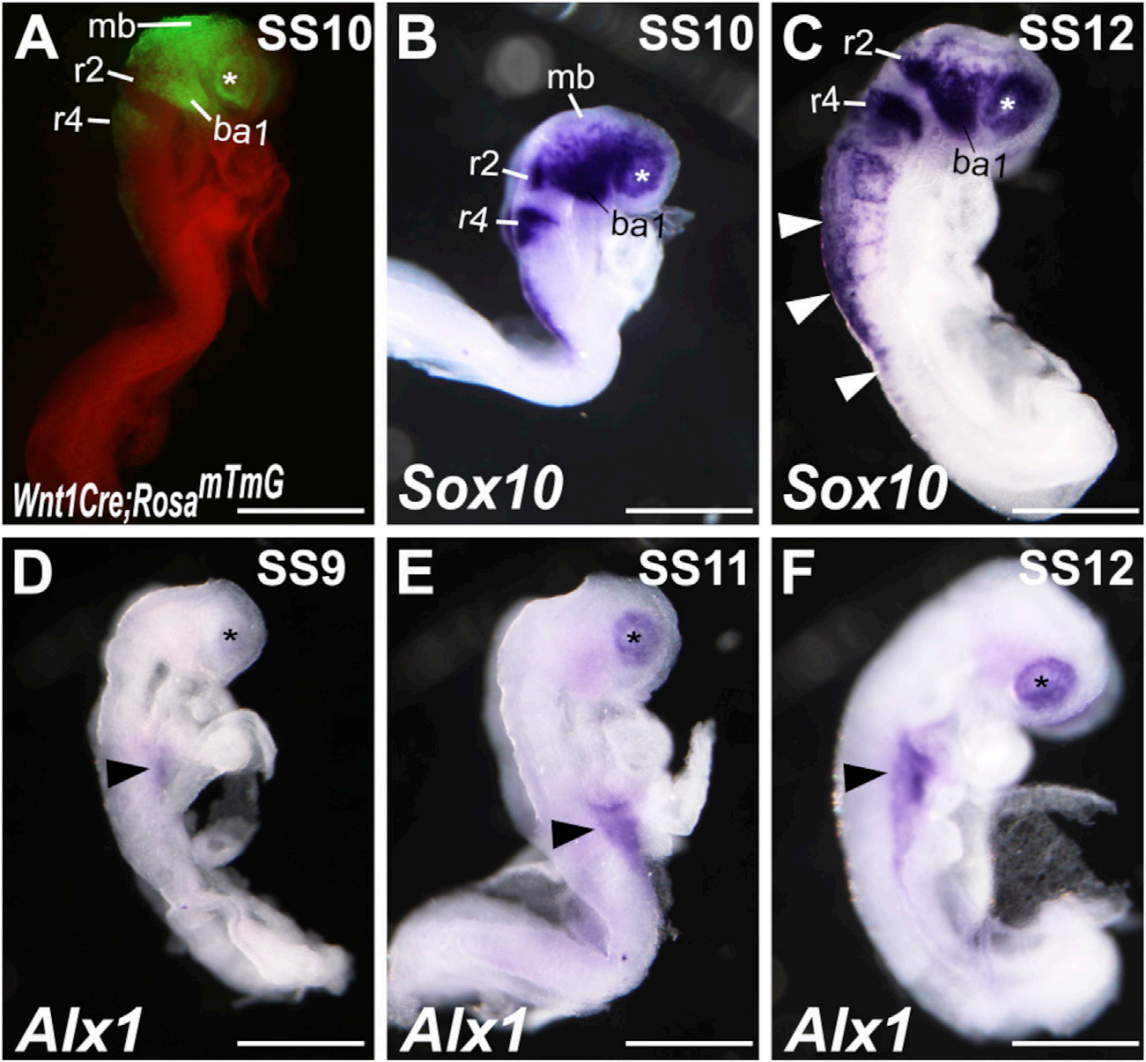
*Alx1* expression during early craniofacial development. **(A)** Lateral view of a *Wnt1-Cre;Rosa26* ^*mTmG*^ embryo at somite stage (SS)10 showing GFP (green) labeled dorsal midbrain neuroepithelium and cranial neural crest cells. **(B, C)** Lateral views of wildtype SS10 **(B)** and SS12 **(C)** embryos showing the patterns of *Sox10* mRNA expression detected by whole mount *in situ* hybridization (blue/purple color). White arrowheads in **(C)** point to the *Sox10* expression in the trunk neural crest cells. **(D–F)** Lateral views of wildtype SS9 **(D)**, SS11 **(E)**, and SS12 **(F)** embryos showing the patterns of *Alx1* mRNA expression detected by whole mount *in situ* hybridization (blue/purple color). The asterisk in A-F mark the location of the optic placode. Arrowheads in **(D–F)** point to the *Alx1* expression in the lateral plate mesoderm. Scale bar, 500 μm. ba1, branchial arch 1; mb, midbrain; r2, rhombomere 2; r4, rhombomere 4.

To understand the cellular mechanism of ALX1 function in frontonasal and ocular development, we analyzed the patterns of *Alx1* mRNA expression during early CNCC development. Previous lineage tracing and single-cell RNA sequencing (scRNA-seq) studies have shown that CNCCs first initiate delamination from the anterior neural plate border at about 4-somite stage (SS4) in mouse embryos and migrate to the facial prominences by SS8–SS10 (Yoshida et al., 2008; Zalc et al., 2021). At SS10, the *Wnt1-Cre;Rosa26* ^*mTmG/+*^ mouse embryos, in which all CNCCs as well as Wnt1-expressing dorsal mid- and hind-brain neuroepithelial cells were genetically labeled by the expression of green fluorescent protein (GFP), clearly showed CNCCs populating the periocular region and the first branchial arches (Figure 3A). *In situ* hybridization analysis also showed strong expression of the *Sox10* mRNAs, a marker of pluripotent neural crest cells, in the CNCCs populating the periocular region and first branchial arches in SS10 wildtype mouse embryos (Figure 3B). By SS12, in addition to expression in the CNCCs, *Sox10* mRNAs were detected in the trunk neural crest cells migrating ventrally from the dorsal neural tube region (Figure 3C). In contrast to the patterns of robust *Sox10* mRNA expression in migrating neural crest cells, the earliest *Alx1* mRNA expression in the cranial region of the mouse embryos was detected in the periocular region from SS9 to SS11 (Figures 3D,E). *Alx1* mRNA expression was not detected in migrating CNCCs lateral to the mid- and hindbrain tissues at these stages, in contrast to the GFP expression pattern in the *Wnt1-Cre;Rosa26* ^*mTmG/+*^ embryos and *Sox10* mRNA expression pattern in the wildtype embryos (compare Figures 3D,E with Figures 3A,B). At SS12, *Alx1* mRNA expression in neural crest cells was still restricted to the periocular region and was not detected in *Sox10*-expressing migrating neural crest cells in either the cranial or trunk regions by *in situ* hybridization analysis (Figure 3F, compared with Figure 3C).

To further clarify the patterns of *Alx1* expression during early CNCC development, we analyzed the scRNA-seq data of early CNCCs harvested from SS4 - SS10 mouse embryos that were recently reported by Zalc et al. (2021). This dataset provides an extremely deep whole transcriptome sequencing of the individual CNCCs around the time of active delamination from the anterior neural plate border and migration towards the early facial primordia, with the median number of over 6,500 detected genes per cell (Zalc et al., 2021). As shown in Supplementary Figure S3A, unsupervised clustering of this early CNCC scRNA-seq dataset clearly clustered the cells into six major groups, identified as the neuroepithelial and pre-delamination CNCC “precursors,” the “migrating CNCCs,” the “post-migratory CNCCs,” cranial “placode” cells, “cranial mesoderm” cells, and “endothelial cells,” respectively, according to their marker gene expression profiles (Supplementary Figures S3A–L; Supplementary Table S1). Whereas high levels of *Sox10* mRNA expression was detected in all migrating and post-migratory CNCCs, while *Foxd3* was highly expressed in the migrating CNCCs but down-regulated in post-migratory CNCCs in these samples (Supplementary Figures S3D,E), *Alx1* mRNA expression was mainly detected in post-migratory CNCCs harvested from SS8 and SS10 embryos, with low level of *Alx1* mRNAs detected in a subset of migrating CNCCs harvested from SS6 embryos (Supplementary Figure S3G). We also analyzed expression of *Alx3* and *Alx4* in this scRNA-seq dataset and found that expression of both *Alx3* and *Alx4* mRNAs was detected in a subset of post-migratory CNCCs harvested from SS10 embryos (Supplementary Figures S3H,I), indicating that expression of both *Alx3* and *Alx4* in the CNCC lineage was activated later than that of *Alx1*. Altogether, while the high sensitivity of the scRNA-seq analysis detected *Alx1* mRNA expression in a subset of migrating CNCCs that was not detected in our *in situ* hybridization analysis, these data consistently demonstrate that *Alx1* expression was activated after the onset of CNCC migration and that *Alx1* exhibited a more restricted pattern of expression than that of *Sox10* in CNCCs during early craniofacial development.

**FIGURE 4:**
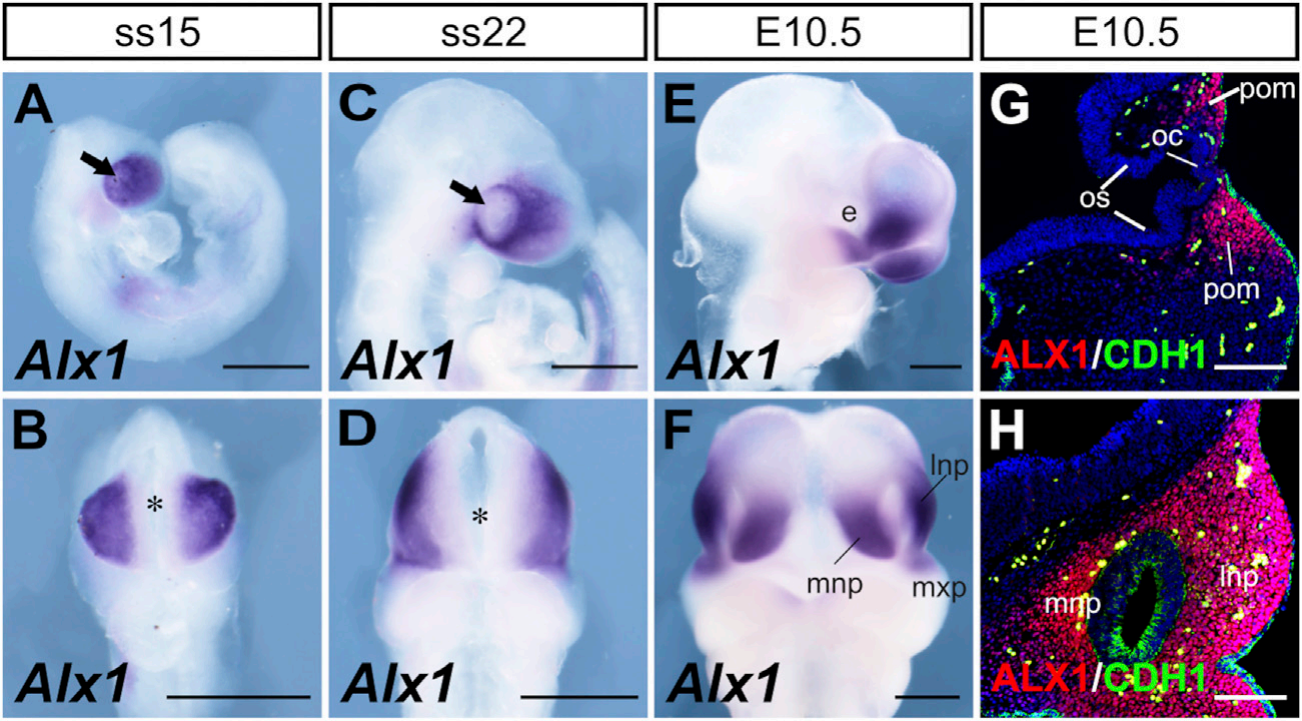
Patterns of expression of *Alx1* during frontonasal development. Whole mount lateral **(A, C, E)** and frontal **(B, D, F)** views of the *Alx1* mRNA expression (blue/purple color) pattern in wildtype embryos at SS15 **(A, B)**, SS22 **(C, D)** and E10.5 **(E, F)**. Arrows in **(A, C)** point to the optic placode. Asterisk in **(B, D)** marks the position of the anterior neural ridge. **(G, H)** Representative frontal sections of E10.5 wildtype embryos showing immunofluorescent staining of ALX1 (red) and CDH1 (E-cadherin) (green) proteins, respectively, in the periocular **(G)** and frontonasal **(H)** tissues. DAPI counterstaining is shown in blue. Scale bars in **(A–F)** and **(G, H)** are 500 and 200 μm, respectively. e, eye; lnp, lateral nasal process; mnp, medial nasal process; mxp, maxillary process; oc, optic cup; os, optic stalk; pom, periocular mesenchyme.

We next analyzed *Alx1* expression during frontonasal development from E8.75 to E10.5 (Figures 4A–D). Frontal views of the embryos showed that *Alx1* mRNAs were concentrated in the lateral regions of the FNP but absent from the anterior midline region overlying the developing forebrain at this developmental stage (Figures 4B,D). At E10.5, strong *Alx1* mRNA expression was detected in the MNP and LNP, with moderate levels of expression also detected in the distal region of the MxP directly adjacent to the LNP (Figures 4E,F). Immunofluorescent staining of serial sections from the E10.5 embryos showed that ALX1 protein was strongly expressed in the CNCC-derived periocular mesenchyme as well as in the mesenchyme of the LNP and MNP, but no ALX1 expression was detected in any of the epithelial tissues, such as the neural epithelium of the brain, optic cup, optic stalk, and the facial and nasal epithelium (Figures 4G,H). These data indicate that ALX1 primarily acts in the CNCCs to regulate frontonasal development.

### Loss of *Alx1* function did not Affect CNCC Migration to the Facial Primordia but Disrupted Optic Stalk Morphogenesis

**FIGURE 5:**
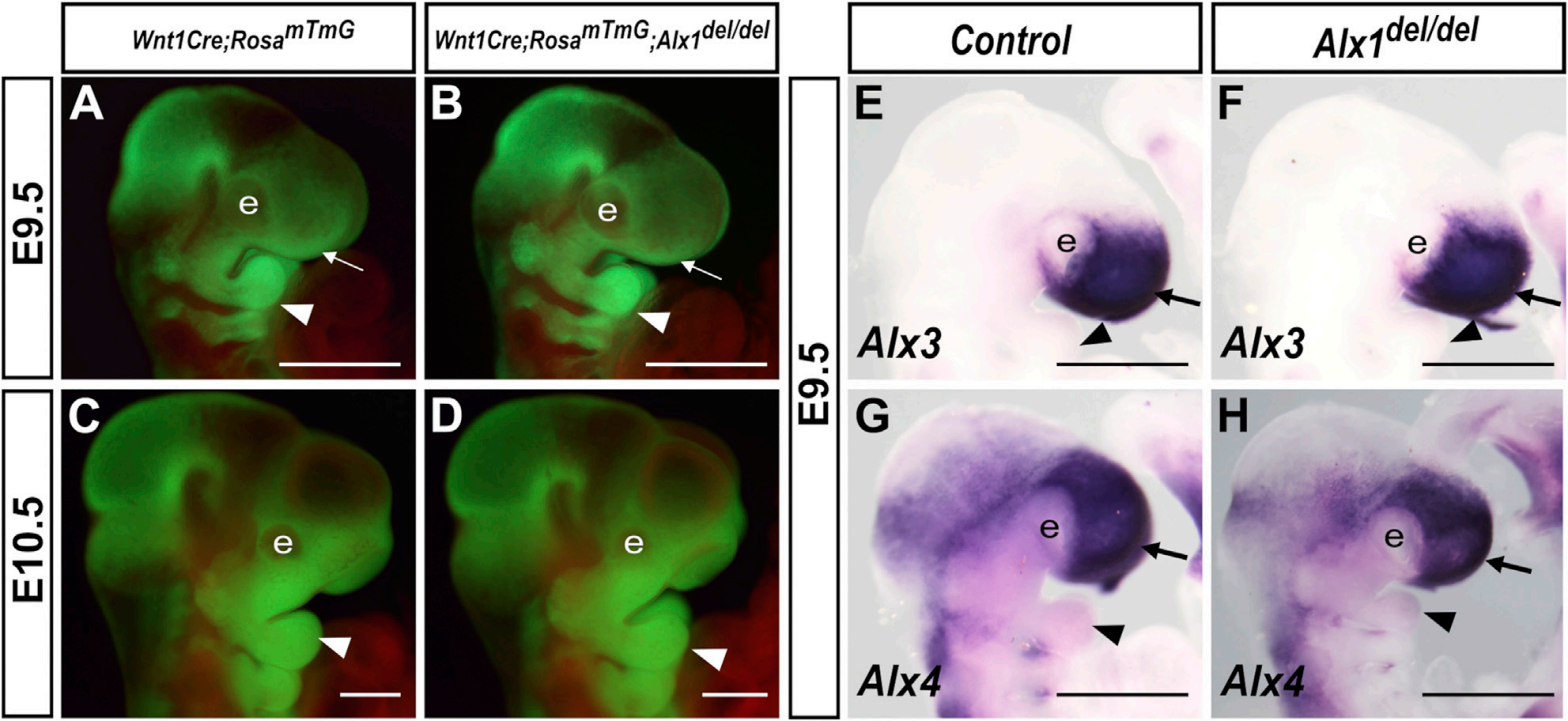
Cranial neural crest migration to the facial primordia and expression of frontonasal neural crest marker genes appeared normal in *Alx1* ^*del/del*^ embryos. **(A–D)** Representative lateral views of whole mount embryo heads showing GFP (green) labeled neural crest cells in the developing facial primordia from *Wnt1-Cre;Rosa26* ^*mTmG/+*^ **(A, C)** and *Wnt1-Cre;Rosa26* ^*mTmG/+*^;*Alx1* ^*del/del*^ **(B, D)** embryos at E9.5 **(A, B)**, and E10.5 **(C, D)**. White arrow in A and B points to the frontonasal prominence, whereas the white arrowhead in **(A–D)** points to the mandibular arch. **(E–H)** Lateral views of whole mount embryo heads showing expression (blue/purple color) of *Alx3* **(E, F)** and *Alx4* **(G, H)** mRNAs in control **(E, G)** and *Alx1* ^*del/del*^ embryos **(F, H)** at E9.5. Arrow in E-H points to the frontonasal prominence, whereas the arrowhead points to the mandibular arch. e, eye. Scale bars, 500 µm.

We next examined whether ALX1 is required for CNCC migration to the facial primordia by using the *Wnt1-Cre:Rosa26* ^*mTmG*^ mediated genetic lineage tracing (Danielian et al., 1998; Muzumdar et al., 2007). As shown in Figure 5, similar patterns of GFP-labeled CNCCs in the frontonasal and periocular regions as well as in the branchial arches were observed in the control and *Alx1* ^*del/del*^ embryos at E9.5 and E10.5 (Figures 5A–D). For embryos examined at E10.5, lateral views of the head consistently detected smaller eyes in the *Alx1* ^*del/del*^ embryos but the contribution of GFP-labeled CNCCs in the nasal, maxillary, and mandibular processes appeared similar in the *Alx1* ^*del/del*^ embryo and control littermates (Figures 5C,D). To further verify that the frontonasal neural crest cells migrated normally to the FNP in the *Alx1* ^*del/del*^ embryos, we analyzed the expression of known frontonasal mesenchyme markers, *Alx3* and *Alx4,* and found that both were similarly expressed in the frontonasal prominence in the control and *Alx1* ^*del/del*^ littermates at E9.5 (Figures 5E–H). These results indicate that early migration of cranial neural crest cells to the FNP was not overtly affected in the *Alx1* ^*del/del*^ mouse embryos, which is consistent with our finding that *Alx1* expression was absent in early migrating CNCCs and was highly expressed in post-migratory periocular CNCCs and the frontonasal mesenchyme.

**FIGURE 6:**
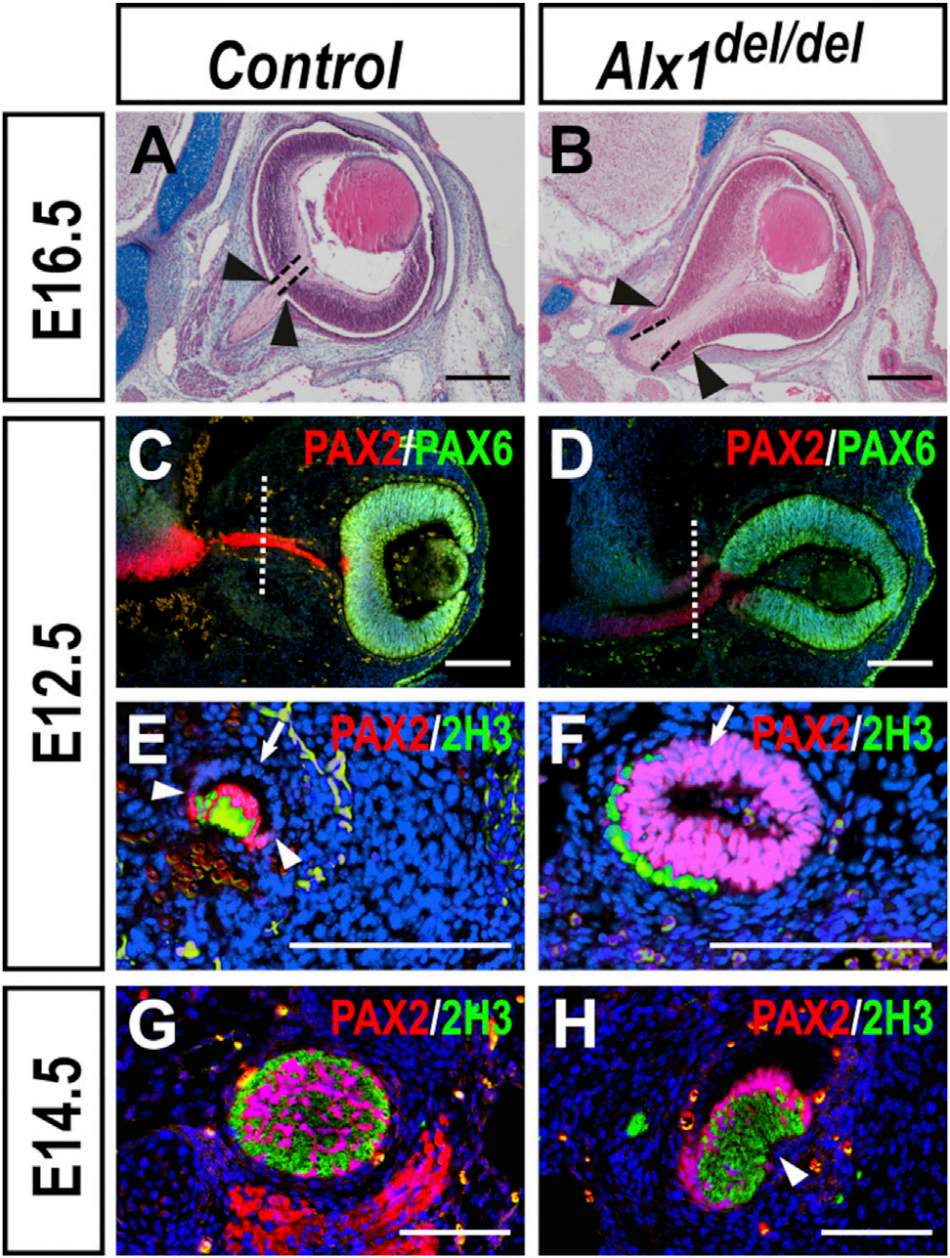
Ocular developmental defects in *Alx1* ^*del/del*^ embryos. **(A, B)** Frontal sections of control **(A)** and *Alx1* ^*del/del*^ **(B)** embryo heads at E16.5. Dashed lines in **(A, B)** mark the exit of the optic nerve from the optic cup, whereas arrowheads point to the proximal boundary of the retinal pigment epithelium. **(C, D)** Immunofluorescent detection of PAX2 (red) and PAX6 (green) in frontal sections of control **(C)** and *Alx1* ^*del/del*^ **(D)** embryo heads at E12.5. White dotted lines in **(C, D)** indicate the corresponding plane of sagittal sections shown in Panels **(E, F)**, respectively. **(E–H)** Immunofluorescent detection of PAX2 (red) and neurofilament in the retinal ganglion axons recognized by the 2H3 monoclonal antibody (green) in sagittal sections through the middle of optic stalk in control **(E, G)** and *Alx1* ^*del/del*^ **(F, H)** embryos at E12.5 **(E, F)** and E14.5 **(G, H)**. White arrowheads in **(E)** point to the boundary of PAX2 expression in the ventral region of the optic stalk epithelium in the control embryo whereas the white arrows in **(E, F)** point to the dorsal region of the optic stalk epithelium. Note that both the PAX2-positive ventral region and the PAX2-negative dorsal/outer layer of the optic epithelium in the E12.5 control embryo **(E)** is 1-2 cell thick and the bilayered optic stalk epithelium wraps around the retinal ganglion axons (green), with the ventral optic fissure still open. In contrast, the optic stalk epithelium in the E12.5 *Alx1* ^*del/del*^ embryo **(F)** is 4-6-cell thick and failed to wrap around the retinal ganglion axons (green). White arrowhead in **(H)** points failure of optic fissure closure in the E14.5 *Alx1* ^*del/del*^ embryo. Scale bars in **(A, B)** and **(C–H)** are 400 and 200 μm, respectively.

Since FND3 patients exhibited extreme microphthalmia and other ocular defects including eyelid coloboma and asymmetric optic nerves (Uz et al., 2010; Pini et al., 2020), we analyzed the ocular developmental defects in *Alx1* ^*del/del*^ embryos. In addition to open eyelids, we found that the *Alx1* ^*del/del*^ pups consistently exhibited smaller eyeballs but the inter-eye distance was not significantly different from wildtype littermates (Supplementary Figures S1D,E). Histological analysis of E16.5 embryos showed that the *Alx1* ^*del/del*^ mutants had deformed optic cup and optic stalk (Figures 6A,B) (*n* = 4 for each genotype). During early eye development, invagination of the optic vesicle results in the formation of asymmetric optic cup with a ventral groove, called the optic fissure, at the ventral side of the optic cup and optic stalk (Tao and Zhang, 2014). From E10.5 to E12.5 in mouse embryogenesis, as the distance between the ventral diencephalon and optic cup increases, the optic stalk epithelium extends along both the medial-lateral and dorsal-ventral axes and changes from an initially 10–12-cell thick neuroblastic epithelial layer to a 1-2-cell thick bilayered epithelial structure around the proximally projected retinal ganglion axons in the optic groove (Evans and Gage, 2005). To better understand the ocular developmental defects in the *Alx1* ^*del/del*^ embryos, we analyzed the patterns of molecular marker expression for the optic cup, optic stalk, and axonal neurofilament in serial sections through the developing optic stalk. At E12.5, while the optic cup and optic stalk neuroepithelia were marked by expression of PAX6 and PAX2, respectively, in both the control and *Alx1* ^*del/del*^ littermates, the optic cup was abnormally extended along the medial-lateral axis in the *Alx1* ^*del/del*^ embryos compared with the control embryos (Figures 6C,D) (*n* = 6 for each genotype). In addition, while the optic stalk epithelium in the E12.5 control embryos had thinned out to a 1–2-cell thick bilayered epithelial structure around the retinal ganglion axons (positive for 2H3 immunostaining) in the optic groove, with only the ventral/inner layer of the optic stalk epithelium expressing PAX2 (Figure 6E) (*n* = 3), the optic stalk epithelium in the *Alx1* ^*del/del*^ littermates remained as an epithelial tube, with a central lumen surrounded by a 4-6-cell thick PAX2+ epithelium, connecting the ventral diencephalon to the optic cup (Figures 6D,F), and with the retinal ganglion axons lying outside of the optic stalk (Figure 6F) (*n* = 3). By E14.5, the PAX2+ cells of the optic stalk had delaminated and integrated with the retinal ganglion axons, forming the organized optic nerve bundle in the control embryos (Figure 6G) (*n* = 3). In the E14.5 *Alx1* ^*del/del*^ embryos, however, the PAX2+ optic stalk cells and the retinal ganglion axons remained largely segregated, with the PAX2+ epithelium partly wrapping around the nerve fibers (Figure 6H) (*n* = 3). These results indicate that ALX1 function is required for optic stalk and optic nerve morphogenesis in mice.

**FIGURE 7:**
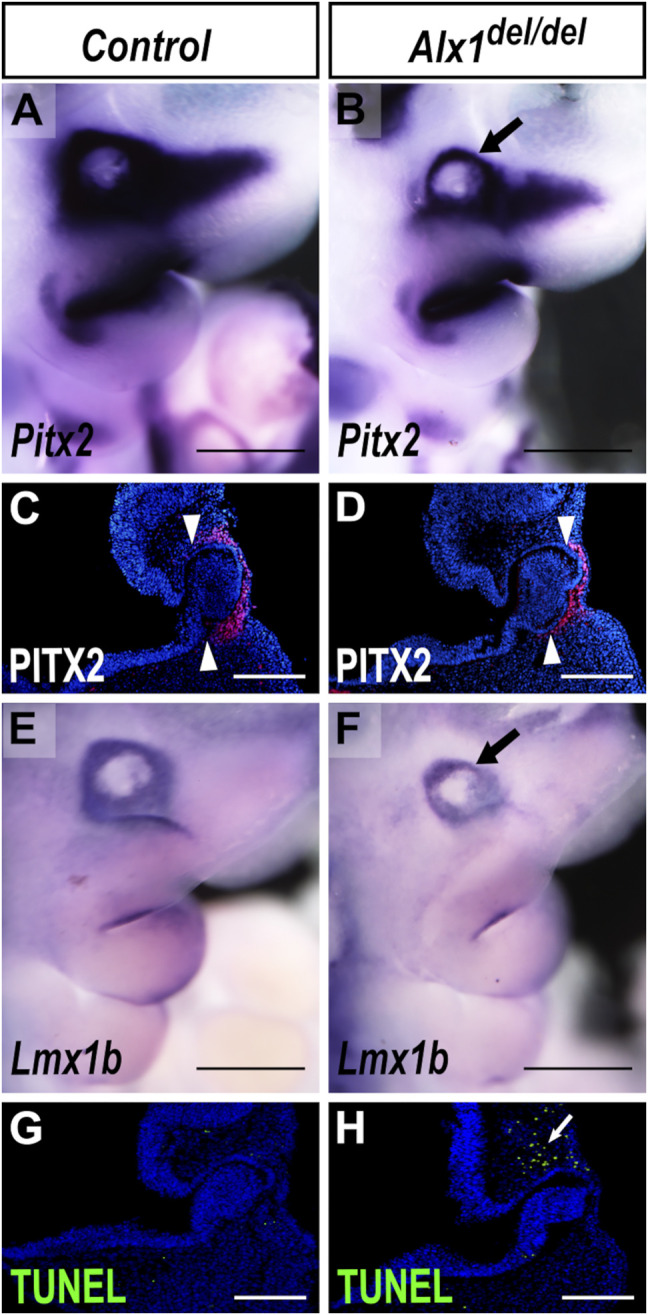
Altered patterns of gene expression and increased apoptosis of the periocular mesenchyme in *Alx1* ^*del/del*^ embryos. **(A, B)** Lateral views of whole mount embryo heads showing *Pitx2* mRNA expression (blue/purple color) in control **(A)** and *Alx1* ^*del/del*^ **(B)** embryos at E10.5. Arrow in **(B)** points to the reduction in the domain of *Pitx2* expression in the periocular region in *Alx1* ^*del/del*^ embryos. **(C, D)** Representative frontal sections of E10.5 embryo heads showing immunofluorescent staining of PITX2 protein (red) in the periocular mesenchyme in wildtype **(C)** and *Alx1* ^*del/del*^ **(D)** embryos. White arrowheads in **(C, D)** point to the restricted domain of PITX2 expression in control and *Alx1* ^*del/del*^ embryos, respectively. **(E, F)** Lateral views of whole mount embryo heads showing *Lmx1b* expression (blue/purple color) in control **(E)** and *Alx1* ^*del/del*^ **(F)** embryos at E10.5. Arrow in **(F)** points to the reduction in the domain of *Lmx1b* expression in the *Alx1* ^*del/del*^ embryo. **(G, H)** Representative frontal sections of E10.5 embryo heads showing TUNEL staining (green) in the periocular mesenchyme in wildtype **(G)** and *Alx1* ^*del/del*^ **(H)** embryos. White arrow in **(H)** points to the domain of cell death detected by TUNEL assay in the *Alx1* ^*del/del*^ embryo. Scale bars in **(A, B, E, F)** are 500 µm. Scale bars in **(C, D, G, H)** are 200 µm.

The defect in optic stalk morphogenesis in the *Alx1* ^*del/del*^ mutants appeared remarkably similar to the optic stalk morphogenesis defect previously reported in mice with neural crest lineage-specific inactivation of *Pitx2* (Evans and Gage, 2005). While *Pitx2* mRNAs were expressed in the periocular mesenchyme surrounding the developing eye in the wildtype embryos at E10.5, expression of *Pitx2* mRNAs in the periocular mesenchyme was apparently reduced in the E10.5 *Alx1* ^*del/del*^ embryos (Figures 7A,B). Immunodetection of PITX2 protein revealed a domain-specific loss of PITX2 protein expression in the periocular mesenchyme surrounding the optic cup (Figures 7C,D). Furthermore, we found that expression of *Lmx1b*, another key ocular developmental regulator (Pressman et al., 2000), was also reduced in the periocular mesenchyme in the E10.5 *Alx1* ^*del/del*^ embryos in comparison with control littermates (Figures 7E,F). Since *Alx1* is expressed in the periocular neural crest cells but not in the optic stalk or optic cup epithelium (Figures 4G,H), these data suggest that ALX1 acts upstream of *Pitx2* and *Lmx1b* in the periocular neural crest cells to regulate eye development. In addition, we found that the *Alx1* ^*del/del*^ embryos consistently exhibited increased apoptosis of the periocular mesenchyme cells located dorsally to the optic cup at E10.5, compared with control littermates (Figures 7G,H) (n = 4 for each genotype). This regional loss of periocular mesenchyme during early eye development likely also contributed to the ocular defects in *Alx1* ^*del/del*^ mice.

### ALX1 Regulates Regional Patterning of the Frontonasal Mesenchyme

**FIGURE 8:**
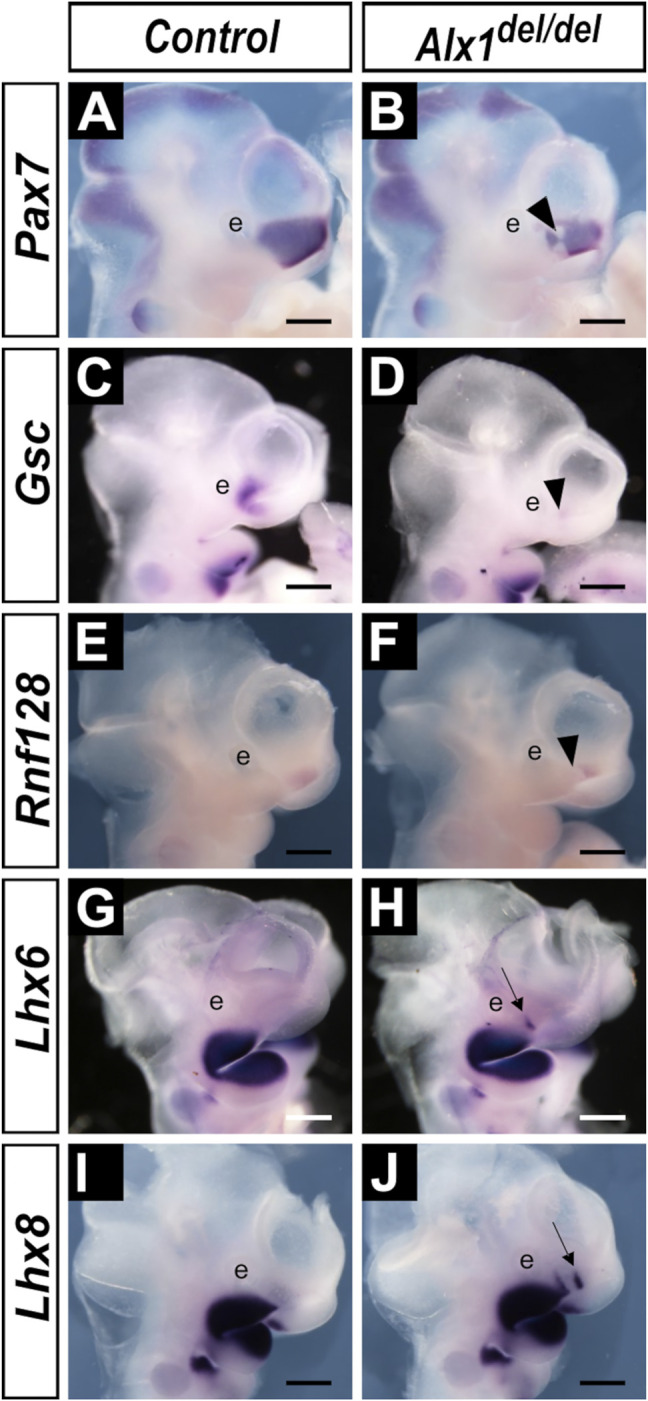
*Alx1* ^*del/del*^ embryos exhibit altered patterns of gene expression in the developing lateral nasal processes. **(A–J)** Lateral views of whole mount embryo heads showing patterns of expression of *Pax7* **(A, B)**, *Gsc* **(C, D)**, *Rnf128* **(E, F)**, *Lhx6* **(G, H)**, and *Lhx8* **(I, J)** mRNAs in the control **(A, C, E, G, I)** and *Alx1* ^*del/del*^ **(B, D, F, H, J)** embryos at E10.5. Arrowhead in **(B, D, F)** points to the domain showing reduction and disruption of the *Pax7, Gsc,* and *Rnf128* expression, respectively, in the *Alx1* ^*del/del*^ embryos. Arrow in **(H, J)** points to the domain of ectopic *Lhx6* and *Lhx8* expression, respectively, in the lateral nasal processes in the *Alx1* ^*del/del*^ embryos. e, eye. Scale bars, 500 µm.

To characterize the developmental mechanism underlying the frontonasal defects in *Alx1* ^*del/del*^ embryos, we compared patterns of expression of several marker genes for distinct regions of the CNCC-derived frontonasal and jaw mesenchyme, respectively. First, we analyzed the expression of *Pax7*, which is critical for nasal cartilage formation (Mansouri et al., 1996). *Pax7* is specifically expressed in the lateral nasal mesenchyme at E10.5 in wildtype embryos (Figure 8A). In the *Alx1* ^*del/del*^ embryos, the *Pax7* expression domain is reduced and is disrupted at the caudal third of the lateral nasal process adjacent to the maxillary process (Figure 8B). Disruption or loss of caudal lateral nasal mesenchyme gene expression was further validated by comparing the patterns of expression of *Gsc* and *Rnf128* in the wildtype and *Alx1* ^*del/del*^ littermates (Figures 8C–F). Next, we analyzed whether the loss of LNP marker gene expression in the *Alx1* ^*del/del*^ embryos reflected a defect in patterning of the facial mesenchyme by comparing the expression of marker genes of the maxillary and mandibular mesenchyme in the *Alx1* ^*del/del*^ embryos and their littermates. Whereas *Lhx6* and *Lhx8* were strongly expressed in the maxillary and mandibular mesenchyme but absent in the lateral nasal mesenchyme in E10.5 wildtype embryos (Figures 8G,I), both *Lhx6* and *Lhx8* mRNAs were found ectopically expressed in the caudal region of the lateral nasal processes in the *Alx1* ^*del/del*^ embryos (Figures 8H,J). These data indicate that ALX1 plays a crucial role in patterning the lateral nasal mesenchyme.

### ALX1 Function in Patterning the Frontonasal Mesenchyme is Partly Complemented by ALX4

Whereas the *Alx1* ^*del/del*^ embryos exhibited evident frontonasal defects including shortened snout, flattened nasal bridge, upper lip notching and premaxillary hypoplasia, *Alx4* ^*−/−*^ mouse embryos showed failure of eyelid closure but relatively normal frontonasal development (Supplementary Figures S4A,D), as previously reported (Qu et al., 1997; Curtain et al., 2015). The *Alx4* ^*-*^ allele (official allele name *Alx4* ^*lst−2J*^) used in this study originally arose in the C57BL/6J inbred background and carries a spontaneous 33.4 kb deletion of the 5′ and exon1 region of the *Alx4* gene (Curtain et al., 2015). In our mouse colony the *Alx4* ^*+/−*^ mice had been previously outcrossed to the wildtype CD1 mice. During this study of *Alx1/Alx4* compound mutants, we examined over 30 *Alx1* ^*del/del*^ embryos generated from *Alx1* ^*del/+*^ *;Alx4* ^*+/−*^ intercrosses and did not detect any phenotypic difference from the *Alx1* ^*del/del*^ embryos analyzed in the C56BL/6 inbred background. However, deleting one allele of *Alx4* in the *Alx1* ^*del/del*^ embryos exacerbated the midfacial phenotype with widened and depressed nasal bridge, shortened philtrum, and further malformed premaxilla in the *Alx1* ^*del/del*^ *Alx4* ^*+/-*^ embryos (Supplementary Figures S4B,E). Furthermore, *Alx1* ^*del/del*^ *Alx4* ^*−/−*^ double homozygous mutants exhibited a wide-open midline facial cleft (Supplementary Figures S4C,F). These data indicate that, while ALX1 function is essential for frontonasal development, ALX4 partly complements ALX1 function in frontonasal morphogenesis.

**FIGURE 9:**
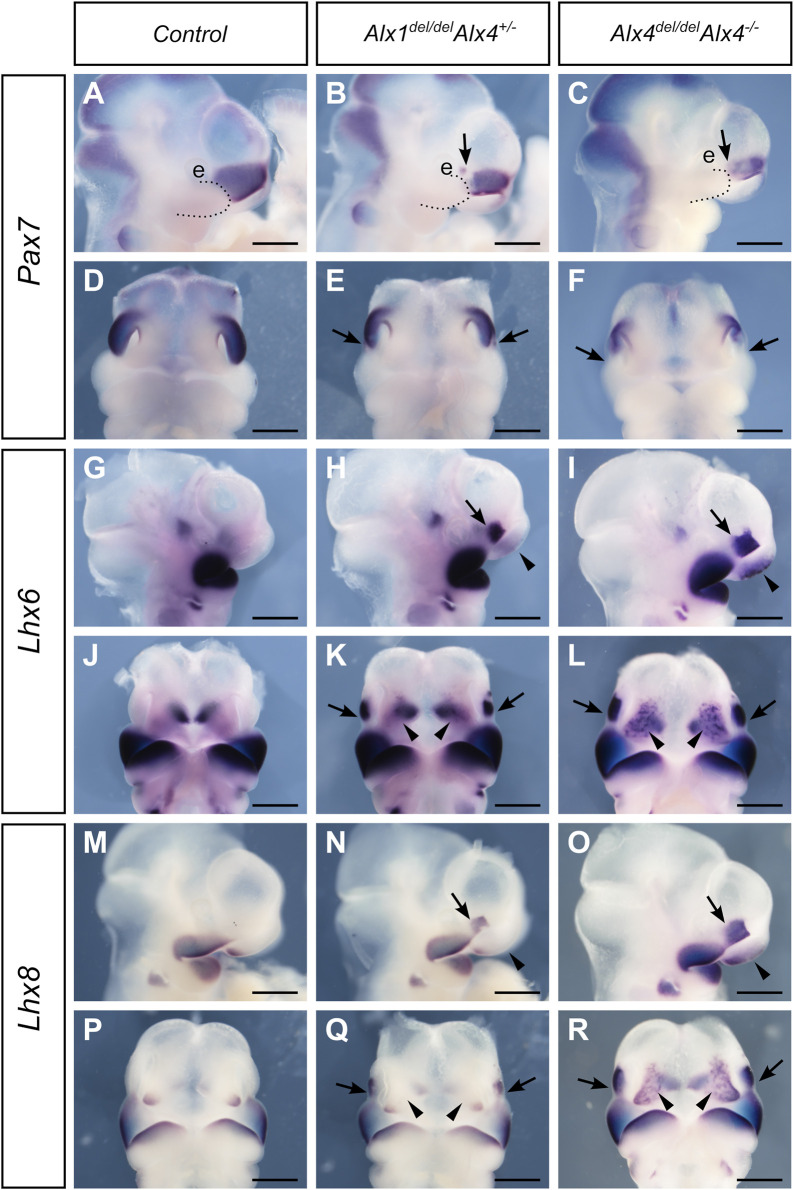
ALX4 partly complements ALX1 function in regulating frontonasal development. Lateral **(A–C)** and frontal **(D–F)** views of whole mount embryo heads showing patterns of *Pax7* mRNA expression in the control **(A, D)**, *Alx1* ^*del/del*^ *Alx4* ^*+/-*^ **(B, E)**, and *Alx1* ^*del/del*^ *Alx4* ^*−/−*^ **(C, F)** embryos at E10.5. Arrow points to the domain of reduced *Pax7* expression in the lateral nasal process of the *Alx1* ^*del/del*^ *Alx4* ^*+/-*^ **(B, E)** and *Alx1* ^*del/del*^ *Alx4* ^*−/−*^ **(C, F)** embryos, respectively. **(G–L)** *L*ateral **(G–I)** and frontal **(J–L)** views of whole mount embryo heads showing patterns of *Lhx6* mRNA expression in the control **(G, J)**, *Alx1* ^*del/del*^ *Alx4* ^*+/-*^ **(H, K)**, and *Alx1* ^*del/del*^ *Alx4* ^*−/−*^ **(I, L)** embryos at E10.5. Arrows and arrowheads point to the domain of ectopic *Lhx6* expression in the lateral and medial nasal processes, respectively, in the in the *Alx1* ^*del/del*^ *Alx4* ^*+/-*^ **(H, K)** and *Alx1* ^*del/del*^ *Alx4* ^*−/−*^ **(I, L)** embryos. **(M–R)** Lateral **(M–O)** and frontal **(P–R)** views of whole mount embryo heads showing patterns of *Lhx8* mRNA expression in the control **(M, P)**, *Alx1* ^*del/del*^ *Alx4* ^*+/-*^ **(N, Q)**, and *Alx1* ^*del/del*^ *Alx4* ^*−/−*^ **(O, R)** embryos at E10.5. Arrows and arrowheads point to the domain of ectopic *Lhx8* expression in the lateral and medial nasal processes, respectively, in the *Alx1* ^*del/del*^ *Alx4* ^*+/-*^ **(N, Q)** and *Alx1* ^*del/del*^ *Alx4* ^*−/−*^ **(O, R)** embryos. e, eye. Scale bars, 500 µm.

We next investigated whether ALX4 plays a complementary role to ALX1 in the patterning of the LNP mesenchyme. Compared with the patchy reduction of *Pax7* expression in the caudal region of the LNP in the E10.5 *Alx1* ^*del/del*^ embryos (Figures 8A,B), E10.5 *Alx1* ^*del/del*^ *Alx4* ^*+/-*^ embryos exhibited a clear loss of *Pax7* expression from the caudal region of the LNP and *Alx1* ^*del/del*^ *Alx4* ^*−/−*^ embryos exhibited a much-reduced domain of *Pax7* expression in the LNP (Figures 9A–F). In a mirror image pattern to the loss of *Pax7* mRNA expression in the caudal region of the LNP, both *Lhx6* and *Lhx8* exhibited enhanced ectopic expression in the caudal region of the LNP in the E10.5 *Alx1* ^*del/del*^ *Alx4* ^*+/-*^ embryos and further expanded domain of ectopic expression in the LNP in the *Alx1* ^*del/del*^ *Alx4* ^*−/−*^ embryos (Figures 9G–R) compared with the patterns of expression in the control and *Alx1* ^*del/del*^ embryos (Figures 8G–J). Furthermore, the *Alx1* ^*del/del*^ *Alx4* ^*−/−*^ embryos exhibited ectopic activation of *Lhx6* and *Lhx8* expression in the MNP as well (Figures 9L,R). These data indicate that ALX1 plays a critical, predominant role in patterning the lateral nasal mesenchyme with ALX4 partly complementing its function for determining the frontonasal mesenchyme identity.

## Discussion

Although a decade has passed since pathogenic mutations in each of the *ALX* family genes *ALX1*, *ALX3*, and *ALX4*, were first reported in FND3, FND1, and FND2 patients, respectively (Kayserili et al., 2009; Twigg et al., 2009; Uz et al., 2010), the cellular and molecular mechanisms involving the ALX transcription factors in frontonasal development remain largely unresolved. FND3 patients exhibited severe frontonasal hypoplasia, microphthalmia, and cleft lip and palate (Uz et al., 2010; Pini et al., 2020), whereas FND1 and FND2 patients displayed milder but distinctive midfacial defects including hypertelorism with ptosis or blepharophimosis, depressed nasal bridge with absent or bifid nasal tip, cleft nasal alae, midline notching of the upper lip, and cranium bifidum (Kayserili et al., 2009; Twigg et al., 2009; Farlie et al., 2016). In this study, we generated mice carrying a deletion of exon-2 of the *Alx1* gene and demonstrated that the *Alx1* ^*del/del*^ mice recapitulate many of the craniofacial defects reported in *ALX*-related FND patients, including eye defects, midfacial hypoplasia with disruption of the nasal cartilages, notching of the upper lip, and cleft palate. Our analyses of the *Alx1* ^*del/del*^ mouse embryos revealed previously unknown roles of ALX1 in regulating regional specification of the CNCC-derived frontonasal mesenchyme. In addition to generating an excellent animal model for understanding the pathogenic mechanisms underlying *ALX1*-related FNDs, these studies provide novel insights into the mechanisms underlying frontonasal patterning during embryonic development of the craniofacial region.

One of the characteristic features of FND is ocular hypertelorism (Sedano et al., 1970; Twigg et al., 2009), also known as orbital hypertelorism, with both terms used to describe widely spaced eyes (Babbs et al., 2011; Sharma, 2014). Ocular hypertelorism is not a disease in and of itself, but rather a physical finding in many craniofacial syndromes, including FNDs and several craniosynostotsis syndromes (Cohen et al., 1995). During prenatal development, the relative position of the eyes changes dramatically, from formation of the optic cup at the lateral sides of the embryonic human head at about 180° from each other in the fifth week of gestation to the frontally located eyes with about a 70° angle between the bilateral optic nerves at the optic chiasm at birth, primarily due to the dramatic growth and expansion of the brain and neurocranium (Cohen et al., 1995). During early postnatal years, although the distance between the orbits increases as the head growth continues, the optic angle further decreases to 68° in adults (Cohen et al., 1995). Compared to humans, the animal models commonly used for craniofacial development studies, including chick and mouse, would be considered naturally extremely hyperteloric as their eyes are laterally positioned. The exact pathogenic mechanisms underlying ocular hypertelorism remain unclear. Several theories of pathogenesis have been suggested, including disruption of nasal capsule formation resulting in the primitive brain vesicle filling the facial midline space and preventing medial migration of the orbits, median facial cleft that disrupts medial integration and causes morphokinetic arrest, early ossification of the lesser wings of the sphenoid bone that fixes the orbits in fetal positions, and premature closure of cranial sutures preventing orbital migration and development (Cohen et al., 1995; Sharma, 2014). The *Alx1* ^*del/del*^ mice exhibited flat and widened nasal bridge, disruption of nasal cartilage formation, notching of the upper lip and cleft palate, but the inter-eye distance was not significantly different from control littermates. The lack of a true ocular hypertelorism phenotype in the *Alx1* ^*del/del*^ mice primarily reflects the differences in the medializing morphogenetic movement of the orbits during mouse and human craniofacial development and does not diminish the value of the *Alx1* ^*del/del*^ mice as an animal model for understanding the molecular and developmental mechanisms underlying FND.

FND3 patients exhibited varying ocular defects including anophthalmia, extreme microphthalmia, and asymmetric optic nerves (Uz et al., 2010; Pini et al., 2020). We found that *Alx1* is expressed in the periocular neural crest cells and not in the optic cup or optic stalk epithelium, but *Alx1* ^*del/del*^ embryos exhibited disruption of optic stalk morphogenesis. The morphological and cellular defects in optic stalk morphogenesis in the *Alx1* ^*del/del*^ embryos were remarkably similar to the optic stalk defects in mouse embryos with neural crest-specific deletion of *Pitx2* (Evans and Gage, 2005). We found that *Pitx2* expression was reduced in the periocular neural crest cells in the *Alx1* ^*del/del*^ embryos at E10.5. Expression of *Lmx1b* was also reduced in the periocular neural crest cells in the *Alx1* ^*del/del*^ embryos, suggesting that *Alx1* is involved in the maintenance or activation of periocular neural crest differentiation program and that the optic stalk defect resulted from a cell non-autonomous function of ALX1 in the periocular neural crest cells.

A previous study of *alx1* morpholino-treated zebrafish embryos showed that *alx1* knockdown inhibited frontonasal CNCC migration and caused catastrophic disruption of facial cartilage formation, which led to the conclusion that ALX1-related FND results from defective CNCC migration (Dee et al., 2013). However, recent reports showed that most *alx1*-null zebrafish mutants developed into adults with no obvious developmental defects (Pini et al., 2020; Mitchell et al., 2021). It has been shown that neural crest migration and survival in zebrafish embryos are susceptible to morpholino-induced artifacts (Boer et al., 2016). Thus, whether ALX1 plays a critical role in CNCC migration requires further validation. Dee et al. (2013) reported that *alx1* expression was activated in the rostral CNCC precursor cells in SS5 zebrafish embryos whereas CNCC migration did not begin until after SS10 (Dee et al., 2013). However, analysis of a recently reported scRNA-seq dataset for early CNCCs from SS4 to SS10 mouse embryos (Zalc et al., 2021) indicates that *Alx1* expression was initially activated in a subset of migrating CNCCs after their onset of migration to the facial primordia. Our analysis of the genetically labeled CNCCs in the *Alx1* ^*del/del*^ and control embryos did not detect any overt changes in CNCC distribution in the early facial primordia at E9.5. However, our results have not ruled out the possibility that loss of function of *Alx1* might have subtle effects on CNCC migration in the periocular or frontonasal regions that requires more sensitive detection methods to uncover. On the other hand, our analyses of the *Alx1* ^*del/del*^ embryos identified a previously unknown role of ALX1 in regulating the regional patterning of the neural crest-derived frontonasal mesenchyme. Whereas *Pax7* and *Rnf128* were specifically expressed in the LNP mesenchyme and *Lhx6* and *Lhx8* were specifically expressed in the maxillary and mandibular mesenchyme in wildtype mouse embryos at E10.5, *Alx1* ^*del/del*^ embryos exhibited a loss of *Pax7* expression and concomitant ectopic expression of *Lhx6* and *Lhx8* in the LNP domain adjacent to the maxillary processes. *Pax7* deficient mice exhibited nasal cartilage and capsule defects (Mansouri et al., 1996). Thus, the loss of *Pax7* expression could account for the defect of nasal cartilage differentiation in the *Alx1* ^*del/del*^ embryos at later stages. Lhx6 and Lhx8 are critical regulators of maxillary, palate, and tooth development (Zhao et al., 1999; Denaxa et al., 2009; Cesario et al., 2015). The ectopic activation of *Lhx6* and *Lhx8* expression in the embryonic LNP likely also contributes to the frontonasal defects in the *Alx1* ^*del/del*^ embryos at later stages.

Previous studies have uncovered molecular mechanisms patterning the CNCCs populating the pharyngeal arches (PA), but very little is known about how the most anterior CNCCs that populate the frontonasal region are patterned. After the neural crest cells arrive at their destined locations, they respond to intrinsic and external signals to adopt their regional identities directed by the expression of combinations of transcription factors (Depew et al., 2002). PA1 and PA2 are defined by a HOX-code, wherein loss of *Hoxa2* function resulted in the loss of PA2 skeletal elements and mirror duplication of PA1 elements (Gendron-Maguire et al., 1993; Rijli et al., 1993). Within PA1, the maxillary-mandibular identity is defined by a DLX-code, wherein loss of *Dlx5/6* function resulted in a homeotic transformation of the mandible into the maxilla (Depew et al., 2002). Here, we show for the first time that ALX1/4 regulates the regional identity of the CNCC-derived frontonasal mesenchyme. Whereas DLX5/6 controls mandibular identity through activation of *Hand2* gene expression (Charité et al., 2001; Sato et al., 2008), our finding that the *Alx1* ^*del/del*^ *Alx4* ^*−/−*^ embryos exhibited dramatically reduced *Pax7* expression in the LNP and concomitant ectopic *Lhx6* and *Lhx8* expression in both the LNP and MNP suggests that the ALX1/4 transcription factors act to regulate frontonasal mesenchyme identity by repressing expression of the jaw developmental regulators including LHX6 and LHX8. Further studies elucidating whether ALX1 directly represses expression of *Lhx6* and *Lhx8* expression and how ectopic *Lhx6/Lhx8* expression contributes to the frontonasal developmental defects in the *Alx1* ^*del/del*^ embryos will lead to clearer understanding of the molecular mechanisms regulating frontonasal development and patterning.

## Materials and Methods

### Generation of *Alx1^del^* Mice

The sgRNA target sites were selected according to the on- and off-target scores from the CRISPR design web tool (http://CRISPOR.org) (Haeussler et al., 2016). The selected sgRNAs, targeting specific sequences in intron-1 (GTA​AGA​TGT​GGG​TGG​TAC​T) and intron 2 (TTA​CTA​AGT​ATA​GGG​ACA​GG) regions, respectively of the *Alx1* gene, were transcribed *in vitro* using the MEGAshorscript T7 kit (ThermoFisher), purified by using the MEGAclear Kit (ThermoFisher) and stored at −80°C. Individual sgRNA was incubated with CAS9 protein (ThermoFisher) at 37°C for 5 min to form the ribonucleoprotein complex and validated in a small batch of mouse zygotes following electroporation, *in vitro* culture to the blastocyst stage, and genotyping. The mixture of sgRNAs (50 ng/μL each) and CAS9 protein (150 ng/μL) was injected into the cytoplasm of fertilized eggs of the C57BL/6N inbred mice using a piezo-driven microinjection technique (Scott and Hu, 2019). Injected eggs were transferred on the same day into the oviductal ampulla of pseudopregnant CD-1 female mice at approximately 25 eggs per recipient. Pups were born and genotyped by PCR to identify founder mice and Sanger sequencing to verify the deletion of exon-2 and flanking sequences of the *Alx1* locus.

All animal work procedures were performed following the recommendations in the Guide for Care and Use of Laboratory Animals by the National Institutes of Health and approved by Institutional Animal Care and Use Committee (IACUC) at Cincinnati Children’s Hospital Medical Center.

### Mouse Breeding and Genotyping PCR

Heterozygous *Alx1* ^*del/+*^ mice were maintained in the C57BL/6N background. Embryos from the *Alx1* ^*del/+*^ intercrosses were collected at respective stages with the noon of the vaginal plug observed as E0.5. *Alx1* ^*del/+*^ intercross embryos were genotyped using the following primers, *Alx1* WT3F: GAA​GCA​TTC​TCA​GCT​AAG​ACT​TG, *Alx1* WT3R: GCA​GTA​TTA​CGT​GCT​GAA​GTG​GT, *Alx1* 3F: GGA​TTC​ATA​CCT​CAT​TGC​AGT​C. For analysis in the 129 x C57BL/6 hybrid background, *Alx1* ^*del/+*^ male mice were bred with 129/S6 inbred females and the female progeny were back-crossed with 129/S6 males to generate N2 males and females, which were intercrossed and embryos were analyzed for skeletal preparations at E18.5 and histology at E16.5.

*The Alx4* ^*lst−2J/+*^ (referred here as *Alx4* ^*+/−*^; Jax Stock #000221) (Curtain et al., 2015) mice were obtained from the Jackson Laboratory. *Alx4* ^*+/−*^ mice had been outcrossed to wildtype CD1 mice in the past and had been maintained by crossing to C57BL/6N mice. For the generation and analysis of *Alx1*/*Alx4* compound mutants, *Alx1* ^*del/+*^ *Alx4* ^*+/−*^ mice were intercrossed, and the embryos dissected at predetermined developmental stages for skeletal preparations or *in situ* hybridization analyses.

### RT-PCR Analysis

PCR primers, forward 5′-GGA​GAC​GCT​GGA​CAA​TGA​GT-3′ and reverse 5′-AGG​CGA​GTG​AGA​GTA​AGG​TG-3′, were used to amplify a 673 bp fragment from exon1 to exon4 of *Alx1* (NM_172553.4). RT-PCR products were gel-purified and sequence-verified at the DNA Core Facility in Cincinnati Children’s Hospital Medical Center.

### Western Blot Analyses

Western blot analyses were carried out as reported previously (Iyyanar and Nazarali, 2017; Okello et al., 2017). In brief, frontonasal and periocular tissues of E11.5 wild-type and *Alx1* ^*del/del*^ embryos, respectively, were lysed in RIPA buffer containing protease inhibitor cocktail. Proteins were quantified using a BCA assay kit (ThermoFisher) and 20 µg proteins were separated on a 4–20% mini-PROTEAN gradient gel (Bio-Rad) and blotted onto a PVDF membrane. Primary antibodies used were anti-ALX1 rabbit polyclonal (1:2000; Proteintech 16372-1-AP) and anti-β-tubulin mouse monoclonal (1:1000; DSHB E7).

### Histology and Skeletal Preparations

For histological analyses, embryos were dissected at desired stages from timed pregnant mice, fixed in 4% paraformaldehyde, dehydrated through an ethanol series, embedded in paraffin, sectioned at 7 μm thickness, and stained with Alcian blue followed by hematoxylin and eosin. Skeletal preparations of E18.5 embryos were processed and stained with Alizarin red and Alcian blue as previously described (Ovchinnikov, 2009).

### Quantitative Measurement of the Midfacial Defects in *Alx1^del/del^* Embryos

Snout length, philtrum length, diameter of the eyeballs, inter-nostril and inter-eye distances were measured using ImageJ software and lateral or frontal view pictures of three pairs of E16.5 control and *Alx1* ^*del/del*^ littermates. Data are represented as mean ± SEM. Statistical analysis was performed by unpaired t-test using the GraphPad Prism software. *p* < 0.05 was considered as statistically significant difference.

### Immunofluorescent Staining and TUNEL Assay

Immunofluorescent staining was performed as previously described (Xu et al., 2016). The following primary antibodies were used: rabbit polyclonal anti-ALX1 (1:200; Proteintech 16372-1-AP), mouse monoclonal anti E-cadherin (BD Biosciences; 610,182), rabbit monoclonal anti-PAX2 (1:400; Abcam Ab79389), mouse monoclonal anti-PAX6 (1:25; DSHB AB_528,427), rabbit monoclonal anti-PITX2 (1:200; Abcam Ab221142), and mouse 2H3 monoclonal anti-neurofilament antibody (1:600; Developmental Studies Hybridoma Bank). Cell death was determined using a TUNEL assay kit in paraffin sections as per manufacturer’s protocol (Promega).

### Whole Mount *in situ* Hybridization

Whole mount *in situ* hybridization was carried out as previously described (Baek et al., 2011). Embryos were staged by counting somite numbers. For each probe analyzed, a minimum of three embryos of each genotype were analyzed and only probes that detected consistent patterns of expression in all samples were considered as valid results. The plasmid templates of the *Alx1*, *Gsc*, and *Rnf128* probes were amplified by PCR and cloned into pBSKII vector using the following primers, *Alx1* F: TAT​ACG​GGG​TTT​TCG​AAC​CA, *Alx1* R: CAC​TCT​GTT​GCA​GCC​TCA​AG; *Gsc* F: CTG​TCC​GAG​TCC​AAA​TCG​CT, *Gsc* R: AGC​ATC​GAC​AAC​ATC​CTG​G; *Pax7* F: GGG​TAG​GGG​GCA​CAG​AGG​CA, *Pax7* R: CCG​GGC​CAG​CAG​GTG​GTT​TC; *Pitx2* F: ACA​TAC​TCA​TAG​ATG​AGA​TG, *Pitx2* R: GAA​ATC​AAA​AAG​GTC​GAG​TT; *Rnf128* F: CAA​CAG​GAC​TGC​CAA​TCA​GG, *Rnf128* R: TGC​ACC​GTA​ACC​AGT​TAC​CAA; *Sox10* F: CGA​AGC​TTC​CAT​CTC​ACG​ACC​CCA​GTT​T, *Sox10* R: CCG​GAT​CCA​GGC​GAG​AAG​AAG​GCT​AGG​T. The *Lhx6* and *Lhx8* (Grigoriou et al., 1998), and *Lmx1b* (Liu and Johnson, 2010) probes were received from published sources.

### Analysis of CNCC scRNA-Seq Data

The scRNA-seq data for the SS4 - SS10 mouse embryonic CNCCs (Zalc et al., 2021) were obtained from the NCBI GEO database (accession number GSE162035). Data analysis was performed using the Seurat package (version 4.0.4, R version 4.0.3) (Hao et al., 2021). Data normalization was performed using the function SCTransform and the heterogeneity associated with ribosomal content and ERCC spike-in content were regressed out. Principal component analysis (PCA) was performed using the function RunPCA. Non-linear dimension reduction was carried out using the function RunUMAP utilizing the first 30 principal components (PCs). The function FindNeighbors was used to construct the Shared Nearest Neighbor (SNN) graph using the first 30 PCs and cell clustering was performed using the function FindClusters with resolution set to 0.2. The cell identities were determined based on their marker genes, which were identified using the FindAllMarkers function.

## Data Availability

The raw data supporting the conclusion of this article will be made available by the authors, without undue reservation.
