# Supplementary material for: Alx1 Deficient Mice Recapitulate Craniofacial Phenotype and Reveal Developmental Basis of ALX1-Related Frontonasal Dysplasia
Source: Front Cell Dev Biol. 2022 Jan 21;10:777887. doi: 10.3389/fcell.2022.777887 (PMC8815032; doi:10.3389/fcell.2022.777887)
Supplement: Supplementary file 1 [file DataSheet1.pdf]

Supplementary Figures for Iyyanar et al, "*Alx1* deficient mice recapitulate craniofacial phenotype and reveal developmental basis of ALX1-related frontonasal dysplasia"

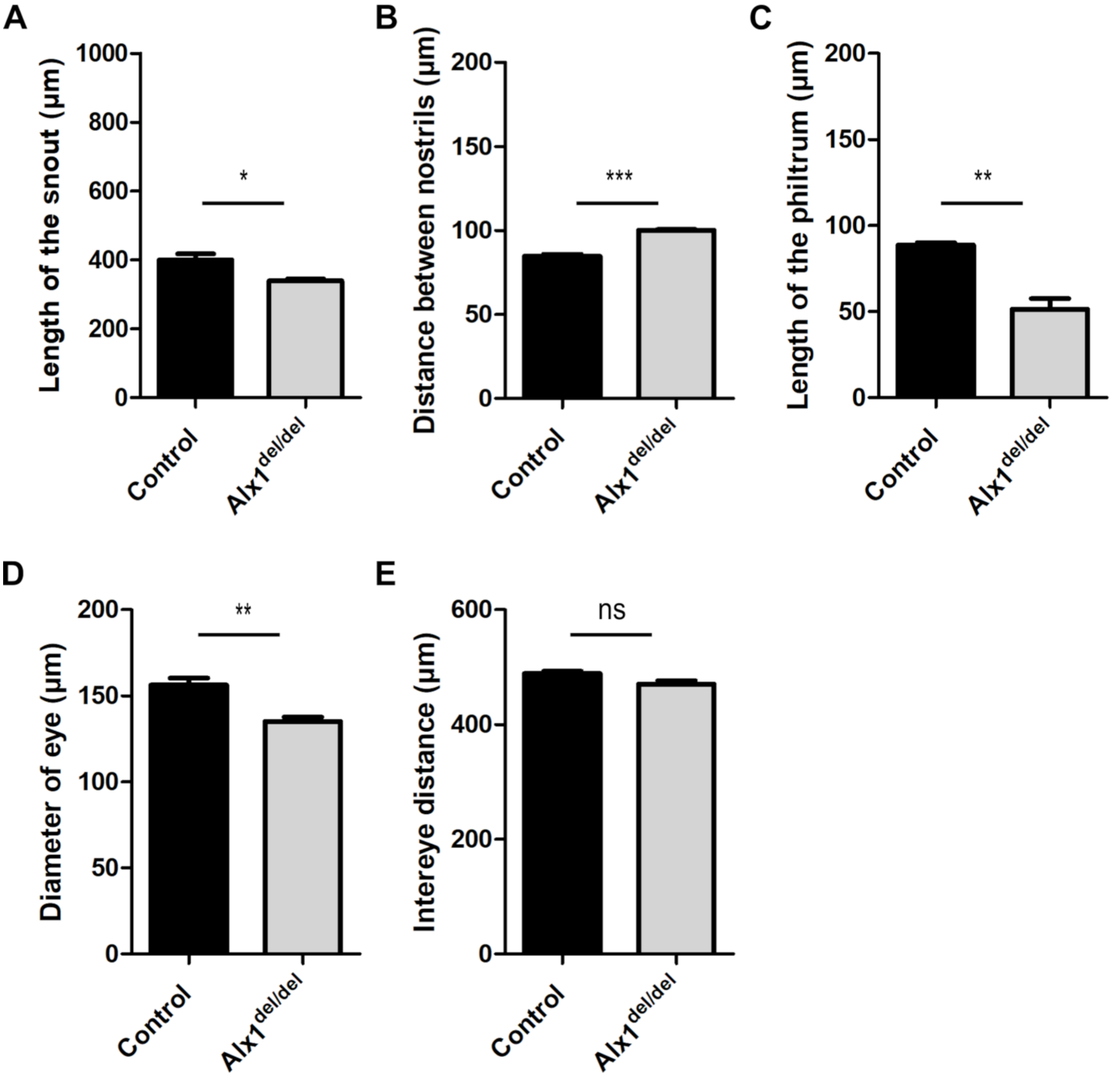

Supplementary Figure S1. Measurements of the frontonasal and eye defects in the *Alx1*$^{del/del}$ embryos at E16.5. (A) Length of the snout measured between the tip of the snout to the distal end of the eye. (B) Distance between the nostrils measured from frontal view pictures of the control and *Alx1*$^{del/del}$ littermates. (C) Vertical philtrum length in the

upper lip from the frontal view pictures of the control and *Alx1*$^{del/del}$ littermates. (D) The horizontal diameter of the eyeball measured from the lateral view pictures (right and left) of the control and the *Alx1*$^{del/del}$ littermates. (E) The distance between the two eyes from the frontal view pictures of the control and *Alx1*$^{del/del}$ littermates. Data were collected from three pairs of control and *Alx1*$^{del/del}$ littermates. All data are represented as mean ± SEM. *, $P < 0.05$; **, $P < 0.01$; ***, $P < 0.001$.

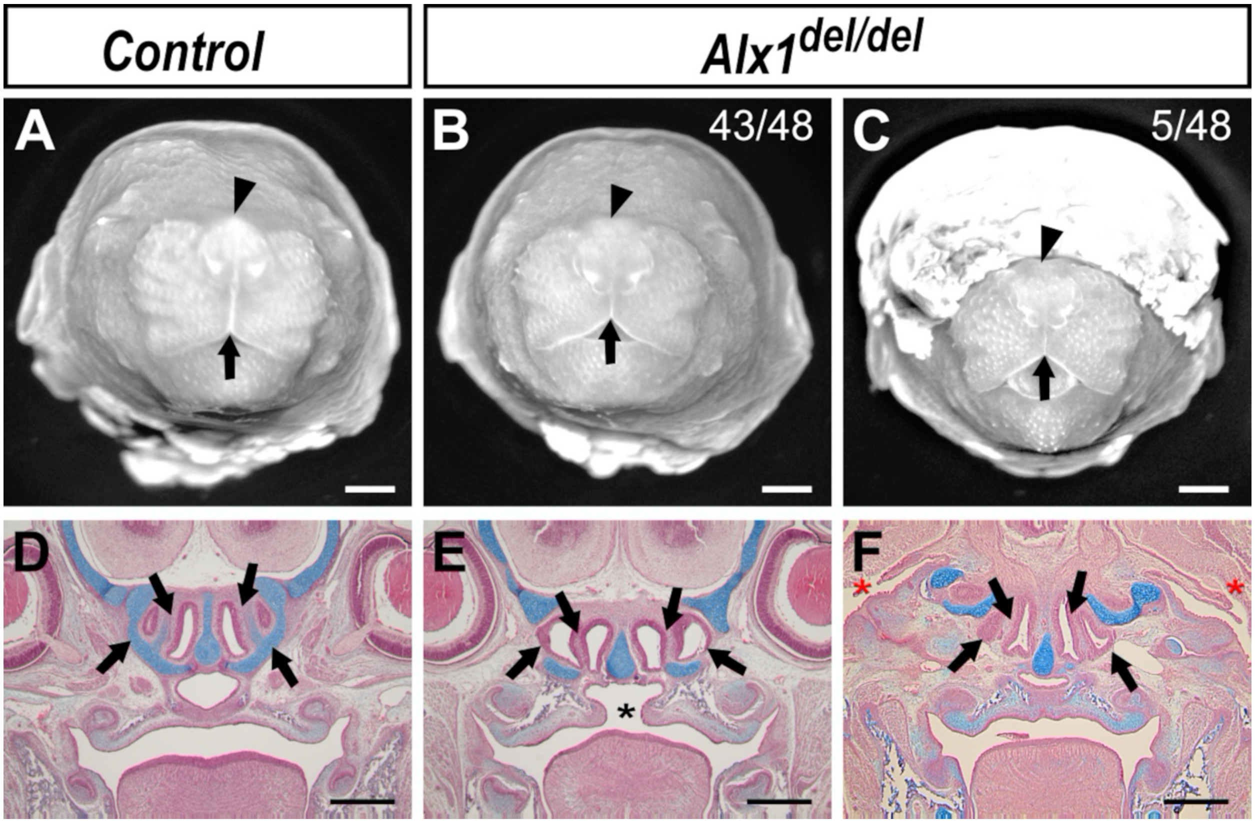

Supplementary Figure S2. Analyses of the craniofacial phenotype in the *Alx1*$^{del/del}$ embryos in the C57BL/6 X 129/S6 hybrid background. (A-C) Frontal view of wildtype control (A) and *Alx1*$^{del/del}$ (B and C) embryo heads at E18.5. Whole mount embryos were stained with DAPI and imaged using a fluorescent stereomicroscope. *Alx1*$^{del/del}$ embryos exhibited flat nasal bridge (arrowhead) and notching of the upper lip (arrow). Numbers in B and C show that 5 of 48 *Alx1*$^{del/del}$ embryos in the hybrid background exhibited exencephaly as shown in C, whereas 43 of 48 *Alx1*$^{del/del}$ embryos in this hybrid background did not have a defect in neural tube closure as shown in B. (D-F) Frontal sections from E16.5 wildtype (D) and *Alx1*$^{del/del}$ embryos without (E) or with (F) exencephaly. Black arrows point to the nasal cartilages in the wildtype (D) and to the

defective nasal cartilages in the *Alx1*$^{del/del}$ embryos (E and F). Black asterisk in E marks the cleft palate. Red asterisks in F mark the absence of eyes. Scale bar, 1 mm.

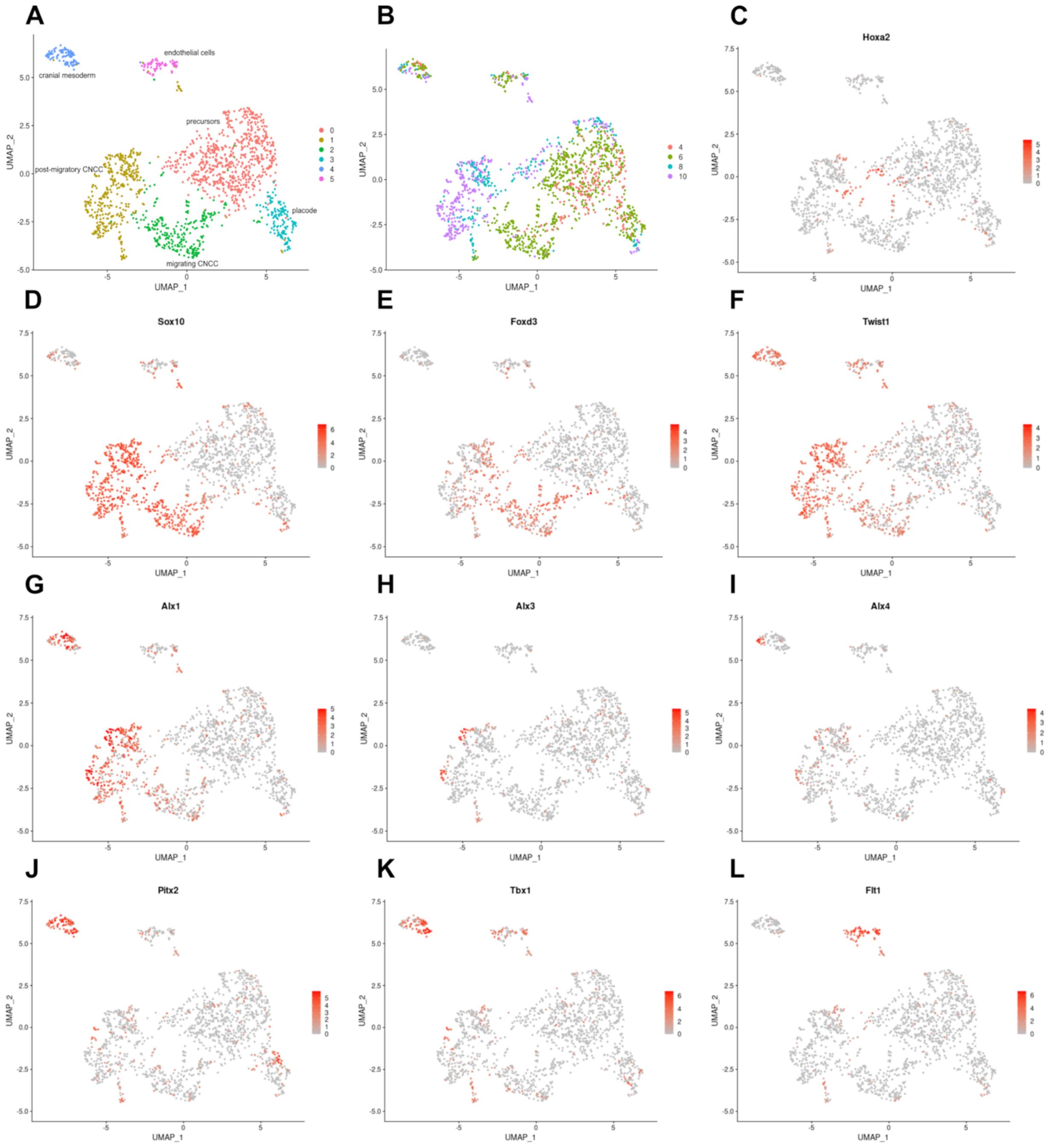

Supplementary Figure S3. Analysis of CNCC gene expression profiles using scRNA-seq data from somite stages SS4 to SS10. (A) UMAP plot showing six annotated major cell clusters. (B) The same UMAP plot but with cells isolated from SS4, SS6, SS8 and SS10 color coded to show the temporal changes in CNCC grouping. Most of the

migrating CNCCs were from SS6 embryos whereas most of the CNCCs from the SS8 and SS10 stages are grouped in the post-migratory CNCCs. (C-L) Visualization of the patterns of expression of the indicated genes using the same UMAP plot. Cells in which at least one unique read was detected for the mRNAs of the indicated gene is shown in red color, with the intensity of the red color reflecting the value of the natural logarithm of the read counts plus 1 (log1p) (darker red color indicates higher level of expression). *Hoxa2* (C) marks neural crest cells posterior to Rhombomere 2. At SS4 to SS10 stages most of the CNCCs were from the forebrain, mid-brain, and anterior hindbrain regions and are *Hoxa2*-negative. *Sox10* (D) and *Foxd3* (E) are markers of multipotent CNCCs, with *Foxd3* down-regulated during ectomesenchyme specification. *Twist1* (F) is upregulated in the ectomesenchyme lineage. Expression of *Alx1* (G) was activated in a subset of migrating CNCCs and upregulated in post-migratory CNCCs whereas expression of *Alx3* (H) and *Alx4* (I) was detected in a subset of post-migratory CNCCs. *Pitx2* (J) and *Tbx1* (K) expression were strong in the cranial mesoderm whereas *Flt1* (L) expression was highly restricted to the endothelial cells.

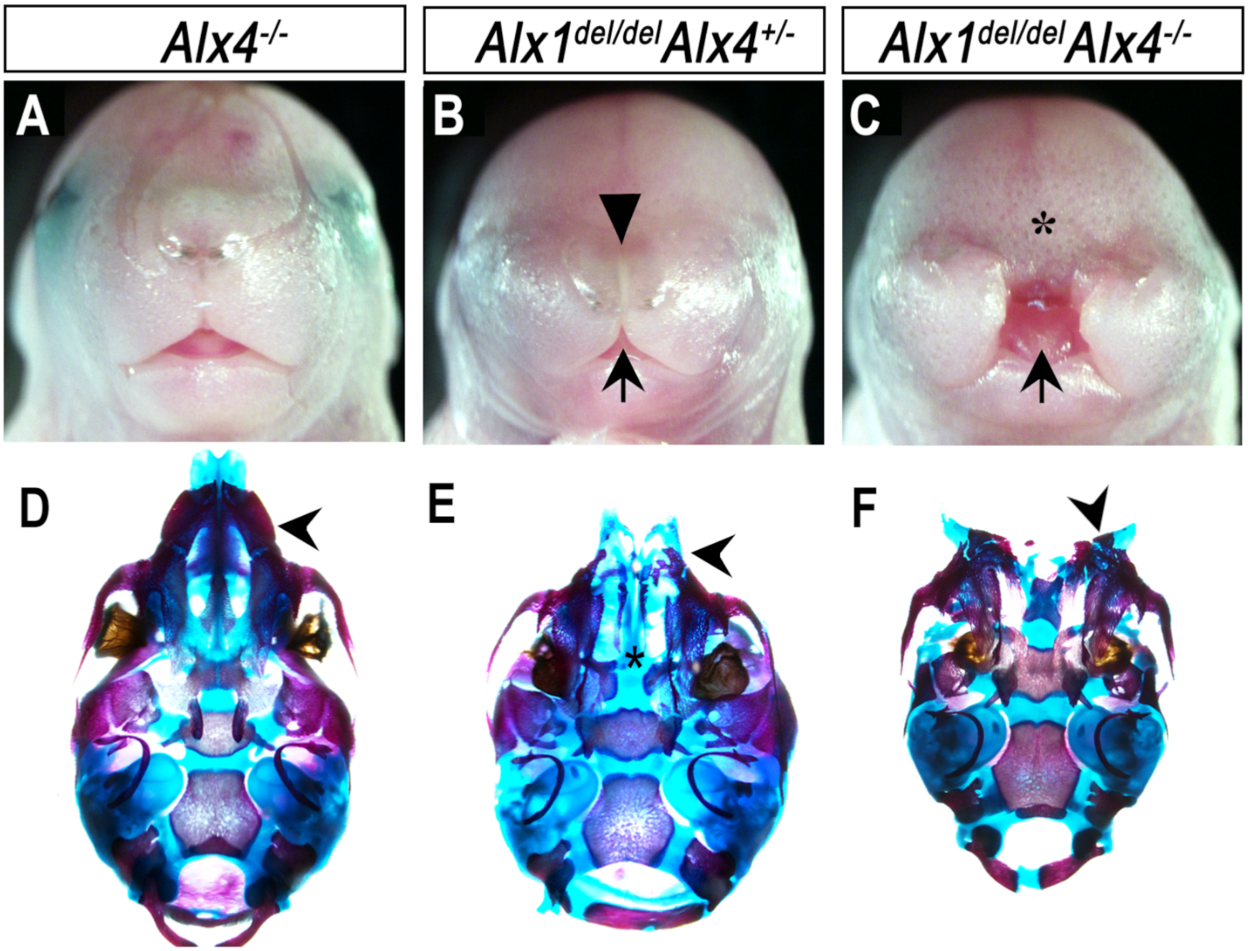

Supplementary Figure S4. ALX4 partly complements ALX1 function in frontonasal development. (A-C) Frontal views of *Alx4*$^{-/-}$ (A) and *Alx1*$^{del/del}$*Alx4*$^{+/-}$ (B) *Alx1*$^{del/del}$*Alx4*$^{-/-}$ embryos at E18.5. While *Alx4*$^{-/-}$ embryos exhibited relatively normal frontonasal development (A), *Alx1*$^{del/del}$*Alx4*$^{+/-}$ embryos exhibited severely depressed and wide nasal bridge (arrowhead in B) and notching of the upper lip (arrow in B). *Alx1*$^{del/del}$*Alx4*$^{-/-}$ embryos exhibited an overt midline facial clefting with a wide gap (asterisk in C) separating the rudimentary nasal structures. Arrow in C points to the wide midline cleft of the upper lip and nose in the *Alx1*$^{del/del}$*Alx4*$^{-/-}$ embryo. (D-F) Palatal view of the head skeleton preparations of *Alx4*$^{-/-}$ (D), *Alx1*$^{del/del}$*Alx4*$^{+/-}$ (E), and *Alx1*$^{del/del}$*Alx4*$^{-/-}$ (F) embryos at E18.5. Arrowhead in D points to the normally developed premaxilla in the *Alx4*$^{-/-}$ embryo,

whereas arrowheads in E and F point to severely malformed premaxilla in the *Alx1*$^{del/del}$*Alx4*$^{+/-}$ and *Alx1*$^{del/del}$*Alx4*$^{-/-}$ embryos, respectively. Asterisk in E marks the malformed presphenoid bone in the cranial base of the *Alx1*$^{del/del}$*Alx4*$^{+/-}$ embryo.
